# Alterations in the elastic properties of the blood clots after the application of the continuous veno-venous hemofiltration in patients submitted to on-pump cardiac surgery

**DOI:** 10.1186/1749-8090-8-S1-O122

**Published:** 2013-09-11

**Authors:** S Pispirigkou, N Theakos, P Dedeilias, A Tsoukas, M Argyriou

**Affiliations:** 1 1st Department of Cardiac Surgery, “Evangelismos” General Hospital, Athens, Greece

## Background

The purpose of this study is to present the changes in the elastic properties of the blood clots and to signify the consequential coagulation disorders after the application of CVVH in patients submitted to on-pump cardiac surgery who presented immediate postoperatively ARF.

## Methods

Over a two-year period (January 2011-January 2013), 32 (3.1%) patients who underwent on-pump cardiac surgery, presented ARF in the immediate postoperative period and submitted to CVVH. Blood samples were obtained in all patients before the start of the CVVH and at least 12 hours after the continuous passage of the blood through the hemofiltration filter of the CVVH-device. All blood samples were tested with thromboelastography in order to identify alterations in the elastic properties of the blood clots induced from the application of the CVVH. The main thromboelastographic parameters recorded were: the reaction time-R, the K-time, the maximum amplitude (MA), the G-index, the Angle and the coagulation index (CI).

## Results

The thromboelastographic parameters R, K, MA, and G were increased from 6.3min, 1.2min, 58.8mm, and 8500d/sc2 to 11.4min (p<0.001), 3.2min (p<0.05), 73.5mm (p<0.01) and 13900 d/sc2 (p<0.001) respectively, while the Angle decreased from 62.7 to 51.8 (p<0.05). The CI was not statistically significant increased from -1.4 to -0.8 (p>0.05).

## Conclusions

The use of the CVVH results to changes of the blood clots elastic properties which are reflected by the increase of the R, K, MA and the G of the formed clot. These changes indicate that the passage of the blood through the hemofiltration filter causes significant disorders in the platelet function and can be applied not only in the patients submitted to cardiothoracic surgery but in all patients subjected to long lasting surgical procedures that present postoperative ARF, as well as in the patients hospitalized in the intensive care units were the application of the CVVH is extremely frequent.

